# Both Central and Peripheral Auditory Systems Are Involved in Salicylate-Induced Tinnitus in Rats: A Behavioral Study

**DOI:** 10.1371/journal.pone.0108659

**Published:** 2014-09-30

**Authors:** Guanyin Chen, Lining Feng, Zhi Liu, Yongzhu Sun, Haifeng Chang, Pengcheng Cui

**Affiliations:** 1 Department of Otolaryngology Head and Neck Surgery, Tangdu Hospital, the Fourth Military Medical University, Xi'an, China; 2 Department of Clinical Medicine, Faculty of Aerospace medicine, the Fourth Military Medical University, Xi'an, China; 3 Department of Radiology, Tangdu Hospital, the Fourth Military Medical University, Xi'an, China; University of Salamanca- Institute for Neuroscience of Castille and Leon and Medical School, Spain

## Abstract

**Objective:**

This study was designed to establish a low dose salicylate-induced tinnitus rat model and to investigate whether central or peripheral auditory system is involved in tinnitus.

**Methods:**

Lick suppression ratio (R), lick count and lick latency of conditioned rats in salicylate group (120 mg/kg, intraperitoneally) and saline group were first compared. Bilateral auditory nerves were ablated in unconditioned rats and lick count and lick latency were compared before and after ablation. The ablation was then performed in conditioned rats and lick count and lick latency were compared between salicylate group and saline group and between ablated and unablated salicylate groups.

**Results:**

Both the R value and the lick count in salicylate group were significantly higher than those in saline group and lick latency in salicylate group was significantly shorter than that in saline group. No significant changes were observed in lick count and lick latency before and after ablation. After ablation, lick count and lick latency in salicylate group were significantly higher and shorter respectively than those in saline group, but they were significantly lower and longer respectively than those in unablated salicylate group.

**Conclusion:**

A low dose of salicylate (120 mg/kg) can induce tinnitus in rats and both central and peripheral auditory systems participate in the generation of salicylate-induced tinnitus.

## Introduction

Tinnitus, a disorder experienced by a significant proportion of the general population [1], [2], is a phantom auditory perception that cannot be attributed to any external sound. The subjective property of tinnitus makes the establishment of an appropriate animal model difficult. Among the medicines that can induce tinnitus in humans as a side-effect, salicylate, an active ingredient of aspirin, is the most prevalently used drug [3], [4]. In establishing a salicylate-induced tinnitus rat model, the rats were conditioned to associate silence and sound respectively with danger and safety in licking water. When salicylate was administrated intraperitoneally (i.p.), a high lick ratio in the silence period would be produced and this high lick ratio is the manifestation of the subjective appearance of sound (tinnitus) [5], [6], [7], [8], [9], [10].

With the prevalent use of the salicylate-induced tinnitus animal models, the process of tinnitus generation induced by salicylate should be explored. Although much progress has been made in understanding the pathophysiological changes of salicylate-induced tinnitus, the mechanisms by which tinnitus is generated are incompletely understood and recent findings are inconsistent. Salicylate can penetrate the blood–brain barrier, thereby providing an opportunity to directly act on the central auditory system [11]. By superfusing central auditory nuclei slices, some studies have reported that salicylate can reduce inhibitory post-synaptic currents [12], [13], [14] and increase the spontaneous activity [15], [16] and these findings are consistent to the results obtained from the systemic application of salicylate [17]. In addition to the in vitro effects salicylate exerts on the central auditory system, several studies have identified that the mechanisms underlying salicylate-induced tinnitus may also involve a peripheral component of the auditory system. The activation of N-methyl-D-aspartate (NMDA) receptors in the spiral ganglion neurons may play an essential role in salicylate-induced tinnitus. Behavioral evidence suggested that the local application of the NMDA antagonist MK-801 to the cochlea could strongly reduce tinnitus generation [7], [18]. Salicylate may inhibit cochlear cyclooxygenase and enable cochlear NMDA receptors, thus leading to elevated activity in the central auditory system. This elevated activity in the central auditory system may be perceived as tinnitus [19]. Salicylate-induced changes in the gene expression in spiral ganglion neurons and in the auditory cortex were reversed by the local application of γ-aminobutyric acid receptor modulator to the cochlea [20]. Moreover, some studies have found that the rate of spontaneous discharge of auditory nerve fibers, a main indicator of the influence of salicylate, was increased after salicylate administration [21], [22]. The findings of all these studies indicate that both central and peripheral auditory systems play a key role in the generation of salicylate-induced tinnitus.

Ablating a possible “tinnitus generator” along the auditory pathway is an important approach to investigating its role in tinnitus generation. Some previous researches have found that in guinea pigs central neuronal activities whose increase levels are correlated to tinnitus reduced immediately after ablation of ispilateral auditory nerve and would not increase before a latency period of at least 3 days [23], [24], indicating that auditory nerve ablation could not produce tinnitus within 3 days after ablation.

This study was designed to establish a tinnitus rat model using a low dose of salicylate and to investigate whether central or peripheral auditory system is involved in salicylate-induced tinnitus by bilateral ablation of auditory nerves in vivo and behavior observation. To discuss the problems, a tinnitus model in conditioned rats was first established using a low dose of salicylate in Experiment One, and the practicability of the lick count and the lick latency, that is, the start time of licking water, during the first 30 s of the first trial was also examined as competent indicators to observe tinnitus performances. The bilateral auditory nerves of unconditioned rats were ablated, and the effect of the ablation itself on rats was observed in Experiment Two. Finally, the effect of salicylate on conditioned rats with bilateral auditory nerves ablation was studied in Experiment Three. We hypothesized that the central auditory system may be involved in salicylate-induced tinnitus because of the possible appearance of tinnitus evidences in the salicylate group compared with the saline group in the ablated rats and the peripheral auditory system may be also involved in salicylate-induced tinnitus because of the probably difficult appearance of tinnitus evidences in the ablated rats compared with the unablated rats in the salicylate groups.

## Materials and Methods

### Animals

Thirty-two male albino Sprague–Dawley rats (280 g to 500 g) were used. Each rat had free access to food but was water-restricted to ∼90% of their pre-deprivation weight. The rats were individually housed on a 12 h/12 h dark/light schedule to maintain their normal biorhythms. The experimental protocol was approved by the Animal Care and Use Committee of the Four Military Medical University. Every effort was made to minimize the use and the discomfort of the rats.

### Measurement of auditory brainstem response

Using a clinical auditory-evoked potential system (Madson Electronics, Copenhagen, Denmark), the threshold recordings of auditory brainstem response (ABR) to click and tone bursts were measured in a sound isolation room. The vertex, bilateral retroauricular areas and snout were depilated using 10% Na_2_S (Sinopharm Chemical Reagent) under anesthesia [30 mg/kg body weight (b. w.) sodium pentobarbital (Merck KGaA, Darmstadt, Germany), i.p.] before the measurement. Rats were fixed in the prone position. ABR signals were detected using three electrodes placed in the vertex, the retroauricular area and the snout respectively for the active electrode, reference electrode and ground electrode. Acoustic stimuli were delivered by a headphone 2 cm away from each ear hole and were averaged 256 times at the rate of 21.1/s. ABR signals were band-pass filtered (100 Hz to 3000 Hz) and were amplified (×10,000). ABR measurement was made in a 10-ms recording window. The threshold recordings of ABR to click and tone bursts started from 90 db sound pressure level (SPL) and 68 db SPL, respectively, and were 5 db SPL steps down to the threshold below which a repeated waveform III was not detected. Rats whose thresholds of click and tone bursts were both <20 db SPL (inclusion criteria) were used in this study.

### Conditioning equipment

**Figure 1:**
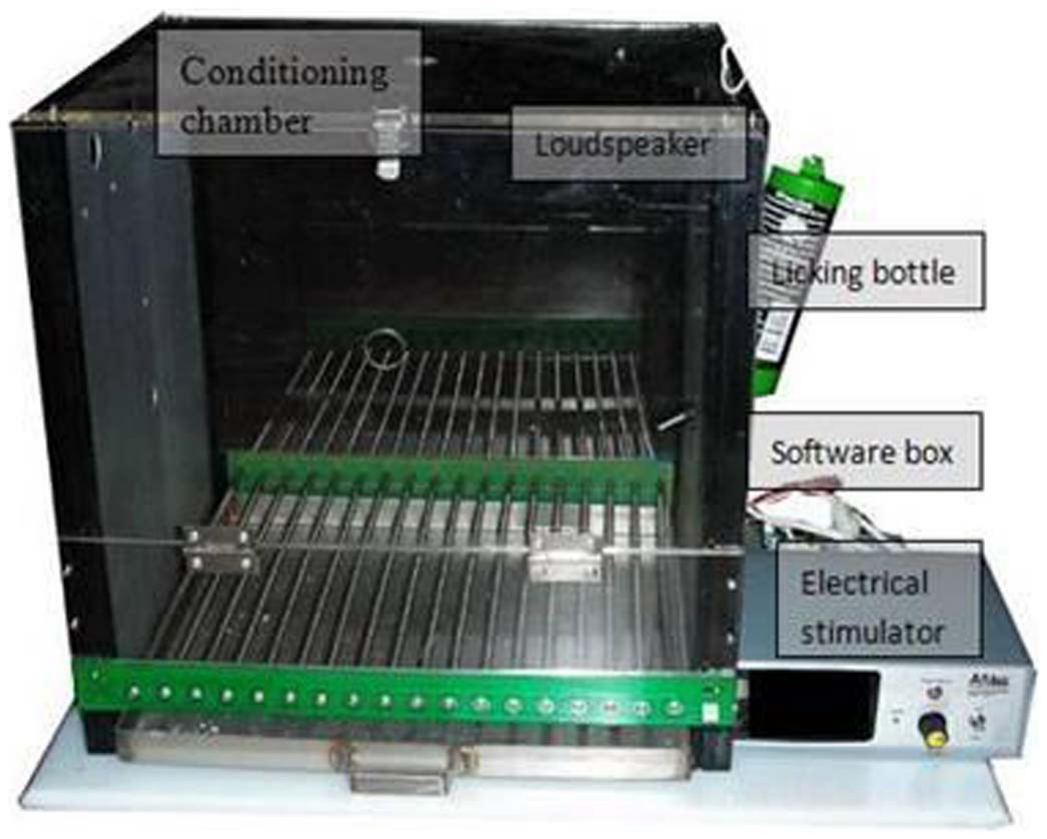
The operant behavioral system used in the study. It includes a conditioning chamber, a licking bottle, an electrical stimulator, and a behavioral software box. The loudspeaker is not shown in the picture.

The operant behavioral system (AniLab Software and Instrument Company Limited, Ningbo, China) was used in the experiment (Fig. 1). The software of the system worked under a Windows operating system [25]. A metal spout of a water bottle was placed through a hole (1 cm diameter) in the wall of the conditioning chamber (0.29 m×0.29 m×0.26 m) and was connected to the anode of an electrical stimulator. The hole was 12 cm away from a stainless steel grid floor which was connected to the cathode of the electrical stimulator. Sound stimuli calibrated with a sound level meter (AR824; Smart Sensor, Hong Kong, China) were generated with the soundcard (SoundMAX) of a computer (Lenovo R60e; Beijing, China), and were delivered to a loudspeaker vertically mounted 10 cm above the hole inside the conditioning chamber. The sampling rate and the bit depth of the soundcard and the loudspeaker are 48 kHz and 16 bit, respectively. Whenever rats licked water, the behavioral system automatically recorded the licking responses and/or delivered a mouth shock (0.2 mA to 0.5 mA; duration, 1 s). The average amount of water delivered on an individual lick was 4 mg-5 mg.

All rats were trained and tested in the same conditioning chamber housed in a sound-attenuating box (0.6 m×0.5 m×0.6 m) lined with sound-absorbing foam. A top-mounted light was used to illuminate the inside of the box, and a USB camera next to the light sent pictures to the computer screen.

### Behavioral protocol

The protocol was based on a classical lick suppression model [5] and was modified for the purpose of this study.

All rats were trained every day in a 15 min session, in which a pure tone (8 kHz, 50 db SPL) was turned off at the first 30 s of the 1st, 5th, 9th and 13th minute. The suspension of the pure tone was paired with a mouth shock whenever rats licked water in the silence period. The lick count recorded in the silence period was indicated by A, whereas the lick count in the subsequent 30 s (the sound period) was signified by B. The suppression ratio R = A/(A+B) in the silence period was thus obtained. Each session was composed of the above four trials and produced 4 Rs. R between 0.4 and 0.6 indicated no difference in the licking responses between the silence and the sound periods, whereas R = 0 indicated the complete suppression of licking water in the silence period. Rats were considered to be conditioned if R<0.2. Before training, rats underwent an acclimation period of 2 days, in which the mouth shock was not used. The purpose of the acclimation was to make rats familiar with the new position of the licking water and to examine whether the pure tone itself had an effect on the licking responses. After acclimation, rats underwent a behavioral training period and then a test period, which lasted 7 and 4 days, respectively. The aim of the behavioral training was to condition the rats to distinguish sound from silence in an approach to associating the sound with licking water (high R in the sound period) and the silence with stopping licking (low R in the silence period). After training, R was not only a running index by which the extent of the behavioral training was obtained, but also an indicator to determine whether the rats had heard sound because high/low R can be explained only by more/less likelihood to hear sound. In the test period, rats in the salicylate group and saline group were injected respectively with 120 mg/kg salicylate (i.p.) and an equivalent volume of saline for 2 consecutive days and then were tested 2 h after injection. No mouth shock was applied in the test period.

### Bilateral auditory nerves ablation

Rats were anesthetized (as described above). Subsequently, auditory nerves were bilaterally ablated, based on the methods described in previous studies [23], [24]. The surgical procedure was applied under aseptic conditions and was carried out visually for ∼1 h at 5× to 10× magnification of a dissecting microscope (Zhenjiang Xin Tian Medical Devices Company, Limited, Nanjing, China). An incision (1.5 cm) was made in the retroauricular area, about 1 cm away from the bottom of the ear. The lateral wall of the bulla was exposed and opened. After puncturing the lateral cochlear wall at the basal turn of the modiolus near the internal auditory canal, the auditory nerve was ablated. The cochlea was packed with absorbable gelatin foam (Jinling Pharmaceutical Company, Limited, Nanjing, China) and the scalp incision was sutured. The auditory nerve on the other side was ablated in the same way. ABR measurement was immediately made after the ablation to ensure that the auditory nerves were fully ablated. Rats were injected with saline (i.p.) after the operation to restore its original body weight (7±0.6 mL) and were allowed 24 h to recover before the test period.

### Experiment One: Effects of salicylate on conditioned rats

To establish a low dose salicylate-induced tinnitus model, fourteen rats underwent acclimation period and training period and ten of them were considered to be conditioned steadily after the training period. They were randomly divided into the salicylate group and the saline group in the test period. The rats were injected respectively with 120 mg/kg salicylate (i.p.) and an equivalent volume of saline for 2 consecutive days and then were tested 2 h after injection for 4 consecutive days in the test period. The lick count and the lick latency during the first 30 s of the first trial and the average R of the four trials in each session were collected during the test period. The weight of water consumption was acquired by recording the increase of the body weight after the test period. In addition, ABR measurement was made after the test period in salicylate group.

### Experiment Two: Effects of bilateral auditory nerves ablation on unconditioned rats

To observe the effects of bilateral auditory nerves ablation on the behaviors of rats, four rats undergoing acclimation period, training period (no mouth shock), ablation surgery, and test period were subjected to Experiment Two. One of them died of bleeding during the surgery. Rats were unconditioned in the training period because no mouth shock existed when rats licked water in the silence period. ABR measurement was made immediately after the ablation. Salicylate or saline was not applied in the test period. The lick count and the lick latency during the first 30 s of the first trial before ablation and for 2 consecutive days after ablation were recorded and compared.

### Experiment Three: Effects of salicylate on conditioned rats with bilateral auditory nerves ablation

To determine the involvement of central auditory system in salicylate-induced tinnitus, sixteen rats underwent acclimation period, training period and ablation surgery. Six of them were excluded either because of unconditioned performance or die of bleeding or anesthesia during the surgery. Ten of them went through the experiment and were randomly divided into the salicylate group and the saline group in the test period after the ablation and ABR was measured. The rats were injected respectively with 120 mg/kg salicylate (i.p.) and an equivalent volume of saline 24 h after the surgery for 2 consecutive days and they were tested in the test period 2 h after injection. The lick count and the lick latency during the first 30 s of the first trial were detected for 2 consecutive days after ablation in the test period. To determine the involvement of peripheral auditory system in salicylate-induced tinnitus, the lick count and the lick latency between the ablated rats in the salicylate group in Experiment Three and the unablated rats in the salicylate group in Experiment One were compared.

### Statistical analysis

SPSS 16.0 version was used in the study. Data were considered to be normal distribution and homogenous variances when *P*>0.1 in Shapiro-Wilk test and Levene's test. Suppression ratio (R) and the water consumption were compared using one-way ANOVA and these data are shown in mean ± standard error (SEM). The lick count and the lick latency were compared using Mann-Whitney U test and these data are shown in median ± quartile range (QR). *P*<0.05 was considered significant.

## Results

### Experiment One: Effects of salicylate on conditioned rats

**Figure 2:**
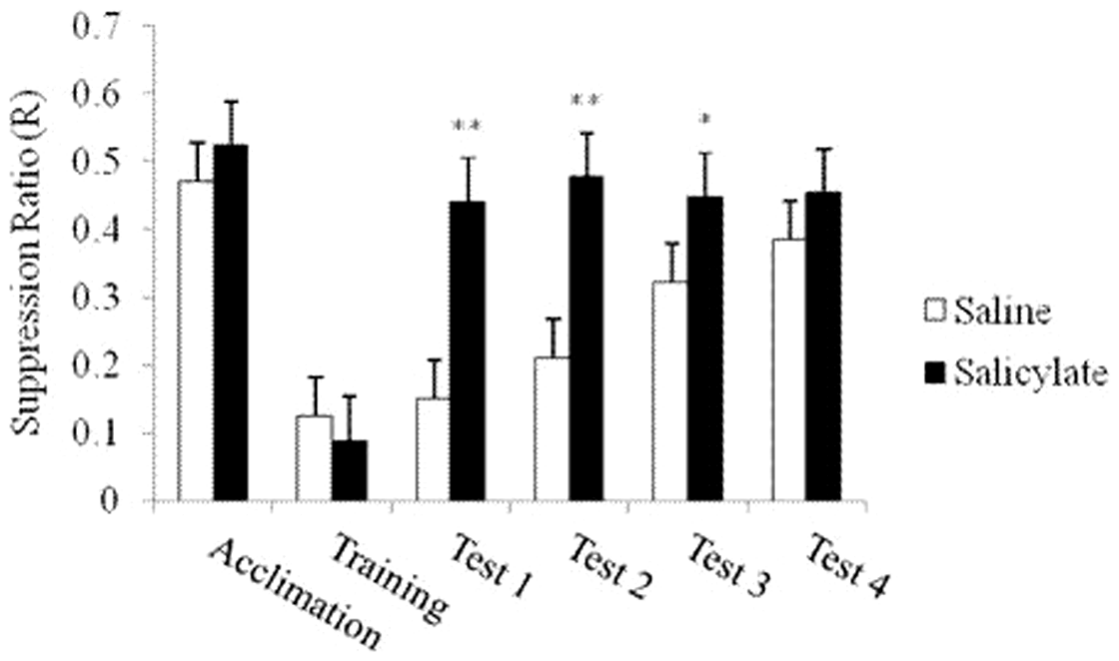
Changes of the suppression ratio (R) in different periods in the saline group and the salicylate group: “Acclimation” shows the average R of all rats in the saline or the salicylate group in the 2-day acclimation period; “Training” shows the average R of all rats in the saline or the salicylate group in the 7-day training period; Tests 1–4 show the 1st, 2nd, 3rd, and 4th day in the test period, respectively. Vertical bars represent mean ± standard error (SEM). **P<0.01 and *P<0.05 are compared with the saline group.

**Figure 3:**
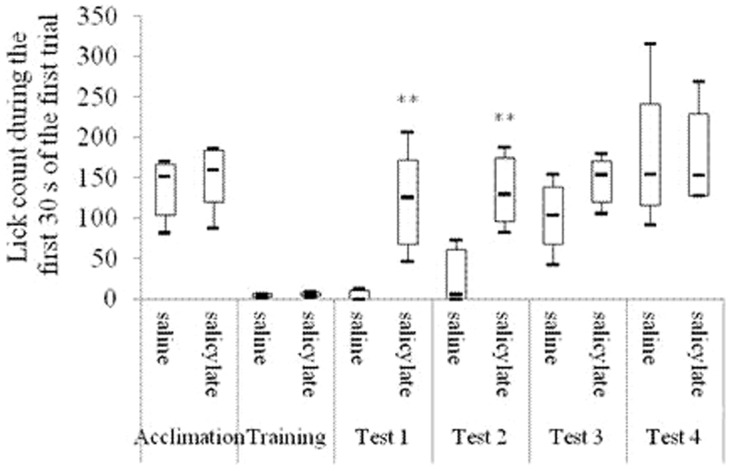
Lick count during the first 30 s of the first trials in different behavioral periods. “Acclimation” shows the average of the lick count of all rats in the saline or the salicylate group in the 2-day acclimation period; “Training” shows the average of the lick count of all rats in the saline or the salicylate group in the 7-day training period; Tests 1–4 show the 1st, 2nd, 3rd, and 4th day in the test period, respectively. **P<0.01 is compared with the saline group.

**Figure 4:**
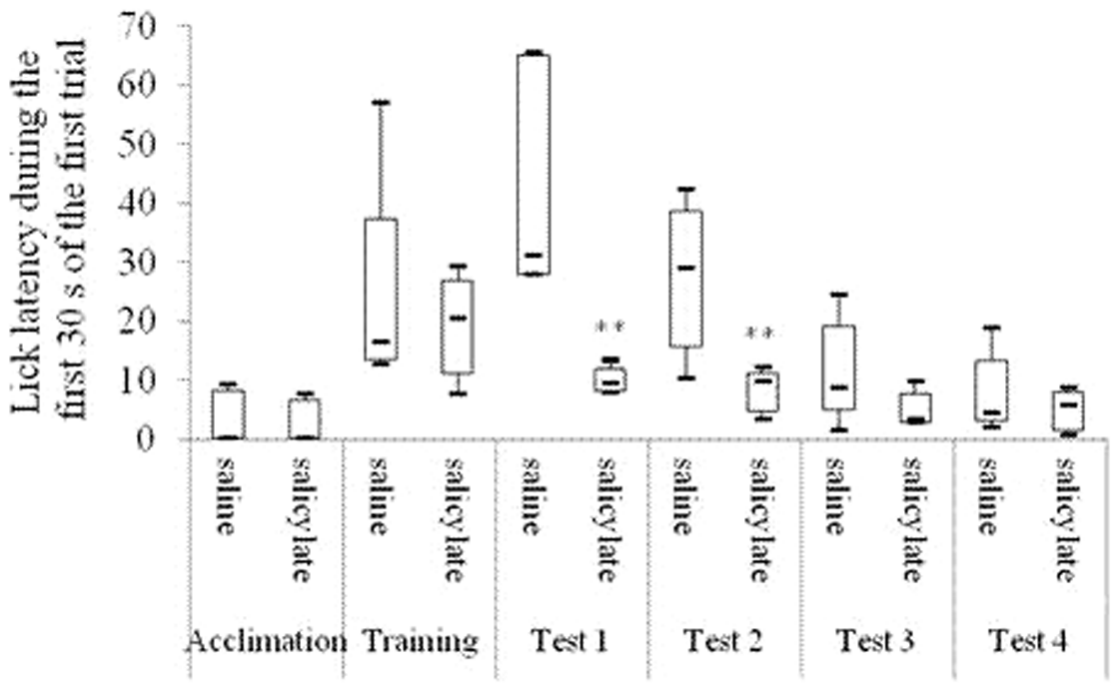
Lick latency (i.e. the start time of licking water) during the first 30 s of the first trials in different behavioral periods. “Acclimation” shows the average of the lick latency of all rats in the saline or the salicylate group in the 2-day acclimation period; “Training” shows the average of the lick latency of all rats in the saline or the salicylate group in the 7-day training period;. Tests 1–4 show the 1st, 2nd, 3rd, and 4th day in the test period, respectively. **P<0.01 is compared with the saline group.

As shown in Fig. 2, both the R values of the salicylate group and the saline group were between 0.4 and 0.6 in the acclimation period. R<0.2 was acquired in the training period. On each of the first 3 test days, the R in the salicylate group was significantly higher than that in the saline group [1st: F(1, 8) = 20.59, P<0.01; 2nd: F(1, 8) = 35.84, P<0.01; 3rd: F(1, 8) = 5.69, P<0.05], but no significant difference was found on the 4th test day [F(1, 8) = 1.05, *P*>0.05]. On each of the first 2 test days, the lick count and the lick latency during the first 30 s of the first trial in the salicylate group was significantly higher [1st: T = 15, P<0.01; 2nd: T = 15, P<0.01] (Fig. 3) and shorter [1st: T = 15, P<0.01; 2nd: T = 16, P<0.01] (Fig. 4) respectively than those in the saline group.

**Figure 5:**
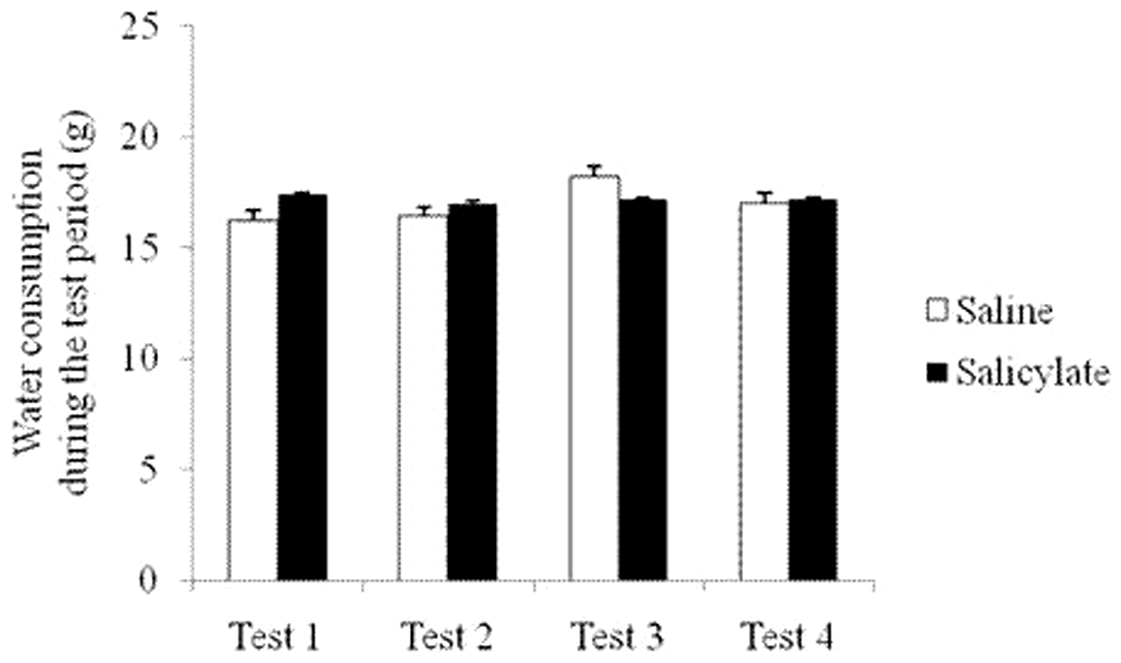
Water consumption during the 4 days in the test period. Tests 1–4 show the 1st, 2nd, 3rd, and 4th day, respectively. Vertical bars represent mean ± standard error (SEM).

No statistically significant differences in the water consumption were found between the saline group and the salicylate group during the test period [1st: F(1, 8) = 0.113, P = 0.745; 2nd: F(1, 8) = 0.056, P = 0.819; 3rd: F(1, 8) = 0.147, P = 0.711; 4th: F(1, 8) = 0.007, P = 0.936] (Fig. 5). ABR thresholds to click and tone bursts did not change before and 2 h after the salicylate administration as shown in Table 1.

**Table 1:** Auditory brainstem response thresholds (db SPL) in different experimental conditions.

	Left ear						Right ear					
Animal number	Before experiment		After salicylate		After surgery		Before experiment		After salicylate		After surgery	
	click	tone	click	tone	click	tone	click	tone	click	tone	click	tone
1-1-1(V)	included 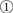						included					
1-1-3(V)	included						included					
1-2-2(V)	included						included					
1-2-8(V)	included						included					
1-2-9(V)	included						included					
1-1-6(S)	5	5	5	5			5	5	5	5		
1-1-8(S)	10	5	10	5			10	5	10	5		
1-1-9(S)	15	5	15	5			10	5	10	5		
1-2-4(S)	5	5	5	5			5	5	5	5		
1-2-6(S)	10	5	10	5			10	5	10	5		
2-5-5(A)	included				>90	>68	included				>90	>68
2-5-6(A)	included				>90	>68	included				>90	>68
2-5-7(A)	included				>90	>68	included				>90	>68
3-1-2(VA)	10	5			>90	>68	10	5			>90	>68
3-1-5(VA)	included				>90	>68	included				>90	>68
3-2-1(VA)	10	5			>90	>68	10	5			>90	>68
3-2-2(VA)	included				>90	>68	included				>90	>68
3-2-5(VA)	included				>90	>68	included				>90	>68
3-1-0(SA)	10	5			>90	>68	10	5			>90	>68
3-2-0(SA)	included				>90	>68	included				>90	>68
3-2-4(SA)	5	5			>90	>68	5	5			>90	>68
3-3-3(SA)	included				>90	>68	included				>90	>68
3-5-1(SA)	included				>90	>68	included				>90	>68

In the column of animal number, the first number stands for Experiment One, Two, and Three, respectively. The last two numbers stand for the serial number of rats used in the study. The following letters “V”, “S”, “A”, “VA”, “SA” in the parentheses stand for the saline group, salicylate group, ablated group, saline+ablated group and salicylate+ablated group, respectively.

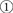
The threshold of click and tone meet the inclusion criteria.

### Experiment 2: Effects of bilateral auditory nerves ablation on unconditioned rats

**Figure 6:**
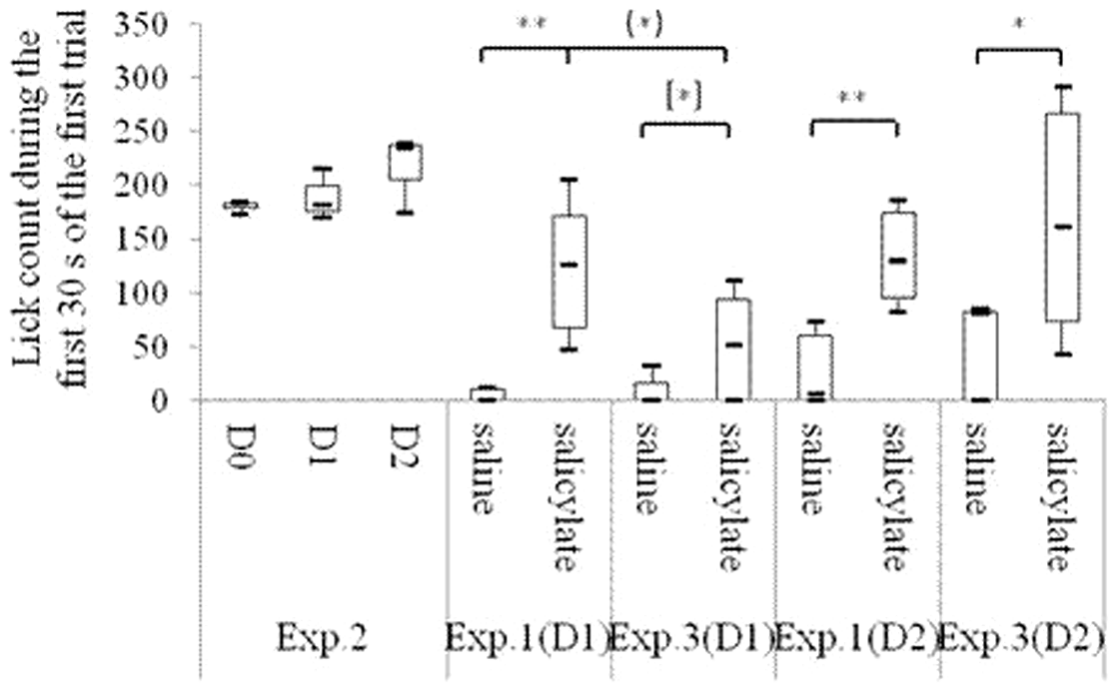
Lick count during the first 30 s of the first trials of Experiment 1, Experiment 2 and Experiment 3. D 0 to D 2 in Experiment 2 indicate the day before operation, the 1st day, and the 2nd day after operation, respectively. D 1 and D 2 in Experiment 1 and Experiment 3 indicate the 1st test day and the 2nd test day, respectively. (*) P = 0.07 for comparison of the salicylate groups between Experiment 1 and Experiment 3. [*] P = 0.1 for comparison between the salicylate group and the saline group in Experiment 3. **P<0.01. *P<0.05.

**Figure 7:**
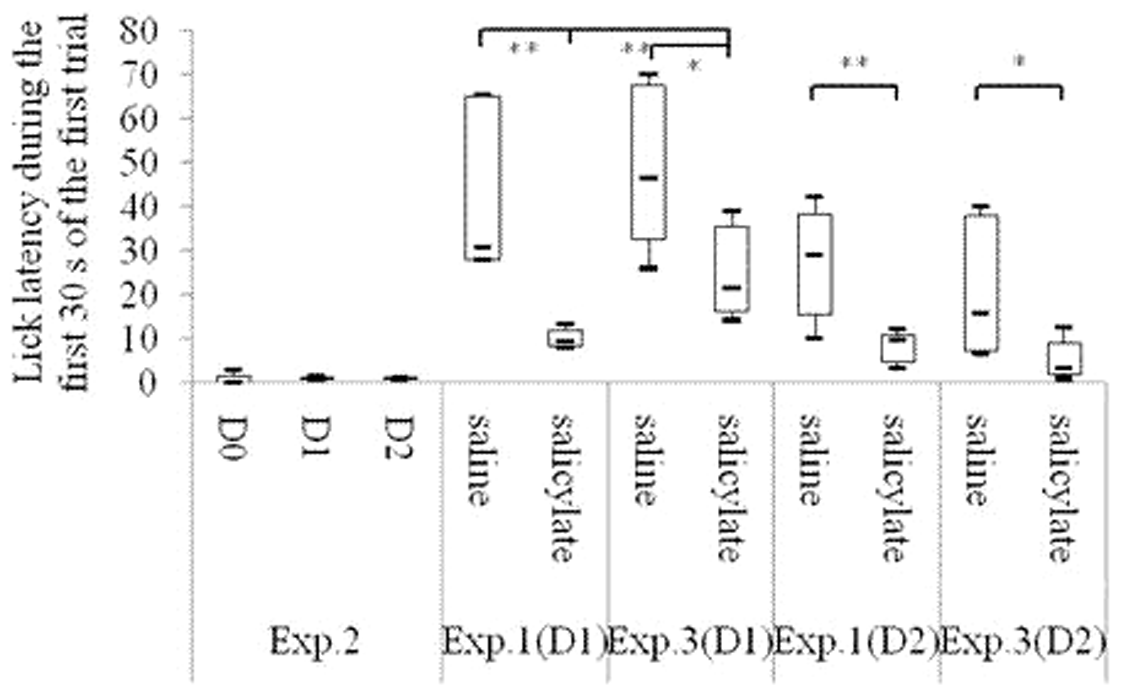
Lick latency (i.e. the start time of licking water) during the first 30 s of the first trials of Experiment 1, Experiment 2 and Experiment 3. D 0 to D 2 in Experiment 2 indicate the day before operation, the 1st day, and the 2nd day after operation, respectively. D 1 and D 2 in Experiment 1 and Experiment 3 indicate the 1st test day and the 2nd test day, respectively. **P<0.01. *P<0.05.

ABR thresholds to click and tone bursts in the bilateral ears of all the rats under study were respectively more than 90 db SPL and 68 db SPL after the ablation as shown in Table 1. During the first 30 s of the first trial on each of the first 2 test days after the ablation, no significant changes compared with those before the ablation were observed in the lick count [1st: T = 10, P = 0.827; 2nd: T = 7, P = 0.127] (Fig. 6) and in the lick latency [1st: T = 9, P = 0.513; 2nd: T = 10, P = 0.827] (Fig. 7).

### Experiment 3: Effects of salicylate on conditioned rats with bilateral auditory nerves ablation

ABR thresholds to click and tone bursts after the ablation were respectively more than 90 db SPL and 68 db SPL in Experiment Three as shown in Table 1. The lick count in the salicylate group during the first 30 s of the first trial was significantly higher than that in the saline group on the 2nd test day [T = 18, P<0.05]. No significant changes were found on the 1st test days [1st: T = 21, P = 0.1] (Fig. 6). The lick latency in the salicylate group during the first 30 s of the first trial was significantly shorter than that in the saline group on the first 2 test days [1st: T = 18, P<0.05; 2nd: T = 17, P<0.05] (Fig. 7). On the first 2 test days, no significant changes were observed in the lick count during the first 30 s of the first trial in the saline groups between Experiment One and Experiment Three [1st: T = 26, P = 0.7; 2nd: T = 23, P = 0.331]. However, the lick count during the first 30 s of the first trial in the salicylate group in Experiment Three was almost significantly lower than that in the salicylate group in Experiment One [T = 19, P = 0.07] (Fig. 6) on the 1st test day. On the first 2 test days, no significant changes were found in the lick latency during the first 30 s of the first trial in the saline groups between Experiment One and Experiment Three [1st: T = 25.5, P = 0.675; 2nd: T = 24, P = 0.465]. However, on the 1st test day, the lick latency during the first 30 s of the first trial in the salicylate group in Experiment Three was significantly longer than that in the salicylate group in Experiment One [T = 15, P<0.01] (Fig. 7).

## Discussion

### Experiment 1: Effects of salicylate administration on conditioned rats

In our study, we used 120 mg/kg salicylate to induce tinnitus in rats. The background sound had no effect on the licking responses in the acclimation period and a stable and qualified R value was produced in the training period. The significantly higher R values in salicylate group indicate that the rats have heard sound (tinnitus) in the silence period and the intensity of tinnitus may be similar to the background sound which was 50 db SPL. Studies have found that salicylate could induce hearing loss and thirst which may result in the high R in the salicylate group [8]. However, no change of the ABR thresholds after salicylate application was observed in our study, indicating no hearing loss existed in the rats of the salicyate group. In addition, no significant difference in the water consumption between the saline group and the salicylate group further shows that subjective sound (tinnitus) is responsible for the high R in the salicylate group. The significantly higher lick count and significantly shorter lick latency during the first 30 s of the first trial suggest that the two indicators could be used in observing salicylate-induced tinnitus.

Many studies have reported that tinnitus can be reliably induced by at least 150 mg/kg salicylate [5], [6], [7], [8], [9], [26]. However, in our experiment we found that 120 mg/kg salicylate can also produce tinnitus in rats. Our finding is consistent with a previous study which found that the minimum dose of salicylate in tinnitus generation was between 75 mg/kg and 150 mg/kg and a 52 db SPL tone may be induced by 150 mg/kg salicylate [27].

### Experiment 2: Effects of bilateral auditory nerves ablation on unconditioned rats

Auditory nerve ablation is a traumatic procedure for rats and may influence their behaviors. ABR measurement showed that the bilateral auditory nerves were completely ablated. No significant changes were found after the ablation in the lick count and the lick latency during the first 30 s of the first trial on the first 2 test days compared with those before the ablation, indicating that the ablation did not significantly affect the behavioral performance of the rats.

It was verified that the metabolic activity marker [14C] 2-deoxyglucose (2DG) was immediately reduced after the ipilateral auditory nerve ablation, and was increased at least 3 days after ablation. The spontaneous increase of metabolic activity was consistent with the findings seen after salicylate administration [24]. Therefore, they suggested that the increased activities may reflect a tinnitus-like phenomenon in guinea pigs. In order to get rid of the interference of the spontaneous increase activities in the auditory pathway after auditory nerve ablation in our study, we recorded and compared the data in 2 days after the ablation.

### Experiment 3: Effects of salicylate on conditioned rats with bilateral auditory nerves ablation

In this experiment, the conditioned rats with bilateral auditory nerves ablation were afraid to lick water at the beginning of the test period but they would be consistent with licking when they started to lick water. This may be explained that there was no external alternative appearance of silence and sound and only silence period was left for the rats with bilateral auditory nerves ablation. Moreover, the rats were conditioned to associate silence with stopping licking water in the training period and no mouth shock existed in the following test period. Therefore, the conditioned rats with permanent hearing loss (similar with the permanent silence period) underwent long lick latency before they started to lick water in the test period and they would lick water continuously when they started to lick. This made their conditioned behavior washed out quickly. Therefore, the data collected from the first 30 s of the first trial was the most meaningful in this experiment and we compared them with those in Experiment One.

The R values in Experiment One were calculated from the lick count under the condition of normal hearing, while the R values after bilateral auditory nerves ablation in Experiment Two and Three were derived from the lick count under the condition of hearing loss. The findings that the lick count and the lick latency during the first 30 s of the first trial in the salicylate group were significantly higher and shorter respectively than those in the saline group in Experiment Three are similar to those in Experiment One. These results suggest that tinnitus can be induced in rats with bilateral auditory nerves ablation in the salicylate group and that a direct pharmacological effect of salicylate on central auditory structures which are unique for the generation of salicylate-induced tinnitus in ablated rats. It has been reported that salicylate may suppress GABAergic and inhibitory neurons and raise excitability in the central system and thus produce tinnitus in in vitro studies by salicylate perfusion of the pyramidal neurons and interneurons of auditory cortex [12], [14]. In addition, salicylate perfusion can increase the spontaneous activities of inferior colliculus and the hyperexcitability may be related to tinnitus [15], [16]. Our in vivo result is the first time to confirm the direct central effect of salicylate in tinnitus generation by behavioral evidences and is similar with these in vitro findings which all reported that the central auditory system may be involved in salicylate-induced tinnitus generation.

Given that the ablation itself had exerted no significant effect on the lick count and the lick latency, the significantly longer lick latency and nearly significant lower lick count in the salicylate group during the first 30 s of the first trial in Experiment Three compared with those in the salicylate group in Experiment One indicate that the peripheral auditory system promotes the generation of salicylate-induced tinnitus in normal hearing rats. It has been conformed that salicylate and mefenamate inhibited cochlear cyclooxygenase and increased levels of arachidonate which potentiated NMDA receptor currents. The increased currents were closely related to the occurrence of tinnitus according to the behavioral evidences of rats [17], [19]. Local cochlear application of the GABA^A^ receptor modulator midazolam reduced the perception of salicylate-induced tinnitus in a rat behavioral modal [20]. In addition, a recent study has found that an altered profile of input from the cochlea could determine the expanded cortical representation of the tinnitus induced by salicylate [28]. Our behavioral finding is supported by these previous reports which observed that salicylate may result in tinnitus by acting on the peripheral auditory system.

There are several limitations in this study. The first limitation is that bilateral auditory nerves ablation is a traumatic procedure for rats although no significant difference existed in lick performance after surgery compared with that before surgery. The second limitation is that the present study has no data concerning the perfusion of brain slices or local cochlear application to show the specific auditory structures of salicylate, although we showed that both the central and peripheral auditory systems are involved into the salicylate-induced tinnitus. The third limitation of the present study is that the number of specimens is not high enough for perfect statistical analyses, although the data showed that the lick latency had significant difference and the similar tendency of lick account. Before our study, we refer to these previous studies in which the number of specimens was similar with us [9], [10], [26].

In conclusion, a tinnitus rat model using a low dose of salicylate (120 mg/kg) is established and the behavioral performances of rats with bilateral auditory nerves ablation show that both the central and peripheral auditory systems participate in salicylate-induced tinnitus. Further studies should be conducted to investigate the specific central auditory structures involved in salicylate-induced tinnitus generation.

## Supporting Information

File S1 Figure S1. Auditory brainstem response (ABR) recording of No. 16 rat after salicylate injection in salicylate group in Experiment One. “L” stands for left ear; “R” stands for right ear; “I” stands for intensity of click or tone; “8K” stands for the frequency of tone is 8000 Hz; “[a. b]”: “a” represents the chronological number of the test and “b” refers to the channel number collected. Figure S2. Auditory brainstem response (ABR) recording of No. 18 rat after salicylate injection in salicylate group in Experiment One. Figure S3. Auditory brainstem response (ABR) recording of No. 19 rat after salicylate injection in salicylate group in Experiment One. Figure S4. Auditory brainstem response (ABR) recording of No. 24 rat after salicylate injection in salicylate group in Experiment One. Figure S5. Auditory brainstem response (ABR) recording of No. 26 rat after salicylate injection in salicylate group in Experiment One. Figure S6. Auditory brainstem response (ABR) recording of No. 16 rat before experiment in salicylate group in Experiment One. Figure S7. Auditory brainstem response (ABR) recording of No. 18 rat before experiment in salicylate group in Experiment One. Figure S8. Auditory brainstem response (ABR) recording of No. 19 rat before experiment in salicylate group in Experiment One. Figure S9. Auditory brainstem response (ABR) recording of No. 24 rat before experiment in salicylate group in Experiment One. Figure S10. Auditory brainstem response (ABR) recording of No. 26 rat before experiment in salicylate group in Experiment One. Figure S11. Auditory brainstem response (ABR) recording of No. 11 rat in saline group in Experiment One. Figure S12. Auditory brainstem response (ABR) recording of No. 13 rat in saline group in Experiment One. Figure S13. Auditory brainstem response (ABR) recording of No. 22 rat in saline group in Experiment One. Figure S14. Auditory brainstem response (ABR) recording of No. 28 rat in saline group in Experiment One. Figure S15. Auditory brainstem response (ABR) recording of No. 29 rat in saline group in Experiment One.
(ZIP)

File S2 Figure S1. Auditory brainstem response (ABR) recording of No. 55 rat after surgery in Experiment Two. Figure S2. Auditory brainstem response (ABR) recording of No. 56 rat after surgery in Experiment Two. Figure S3. Auditory brainstem response (ABR) recording of No. 57 rat after surgery in Experiment Two. Figure S4. Auditory brainstem response (ABR) recording of No. 55 rat before experiment in Experiment Two. Figure S5. Auditory brainstem response (ABR) recording of No. 56 rat before experiment in Experiment Two. Figure S6. Auditory brainstem response (ABR) recording of No. 57 rat before experiment in Experiment Two.
(ZIP)

File S3 Figure S1. Auditory brainstem response (ABR) recording of No. 10 rat after surgery in salicylate group in Experiment Three. Figure S2. Auditory brainstem response (ABR) recording of No. 20 rat after surgery in salicylate group in Experiment Three. Figure S3. Auditory brainstem response (ABR) recording of No. 24 rat after surgery in salicylate group in Experiment Three. Figure S4. Auditory brainstem response (ABR) recording of No. 33 rat after surgery in salicylate group in Experiment Three. Figure S5. Auditory brainstem response (ABR) recording of No. 51 rat after surgery in salicylate group in Experiment Three. Figure S6. Auditory brainstem response (ABR) recording of No. 10 rat before experiment in salicylate group in Experiment Three. Figure S7. Auditory brainstem response (ABR) recording of No. 20 rat before experiment in salicylate group in Experiment Three. Figure S8. Auditory brainstem response (ABR) recording of No. 24 rat before experiment in salicylate group in Experiment Three. Figure S9. Auditory brainstem response (ABR) recording of No. 33 rat before experiment in salicylate group in Experiment Three. Figure S10. Auditory brainstem response (ABR) recording of No. 51 rat before experiment in salicylate group in Experiment Three. Figure S11. Auditory brainstem response (ABR) recording of No. 12 rat after surgery in saline group in Experiment Three. Figure S12. Auditory brainstem response (ABR) recording of No. 15 rat after surgery in saline group in Experiment Three. Figure S13. Auditory brainstem response (ABR) recording of No. 21 rat after surgery in saline group in Experiment Three. Figure S14. Auditory brainstem response (ABR) recording of No. 22 rat after surgery in saline group in Experiment Three. Figure S15. Auditory brainstem response (ABR) recording of No. 25 rat after surgery in saline group in Experiment Three. Figure S16. Auditory brainstem response (ABR) recording of No. 12 rat before experiment in saline group in Experiment Three. Figure S17. Auditory brainstem response (ABR) recording of No. 15 rat before experiment in saline group in Experiment Three. Figure S18. Auditory brainstem response (ABR) recording of No. 21 rat before experiment in saline group in Experiment Three. Figure S19. Auditory brainstem response (ABR) recording of No. 22 rat before experiment in saline group in Experiment Three. Figure S20. Auditory brainstem response (ABR) recording of No. 25 rat before experiment in saline group in Experiment Three.
(ZIP)

Data S1 Raw data of suppression ratio (R), water consumption, lick count and lick latency of Experiment One, Experiment Two and Experiment Three.
(XLSX)
