# Supplementary material for: Both Central and Peripheral Auditory Systems Are Involved in Salicylate-Induced Tinnitus in Rats: A Behavioral Study
Source: PLoS One. 2014 Sep 30;9(9):e108659. doi: 10.1371/journal.pone.0108659 (PMC4182535; doi:10.1371/journal.pone.0108659)
Supplement: File S1 — Figure S1. Auditory brainstem response (ABR) recording of No. 16 rat after salicylate injection in salicylate group in Experiment One. “L” stands for left ear; “R” stands for right ear; “I” stands for intensity of click or tone; “8K” stands for the frequency of tone is 8000 Hz; “[a. b]”: “a” represents the chronological number of the test and “b” refers to the channel number collected. Figure S2. Auditory brainstem response (ABR) recording of No. 18 rat after salicylate injection in salicylate group in Experiment One. Figure S3. Auditory brainstem response (ABR) recording of No. 19 rat after salicylate injection in salicylate group in Experiment One. Figure S4. Auditory brainstem response (ABR) recording of No. 24 rat after salicylate injection in salicylate group in Experiment One. Figure S5. Auditory brainstem response (ABR) recording of No. 26 rat after salicylate injection in salicylate group in Experiment One. Figure S6. Auditory brainstem response (ABR) recording of No. 16 rat before experiment in salicylate group in Experiment One. Figure S7. Auditory brainstem response (ABR) recording of No. 18 rat before experiment in salicylate group in Experiment One. Figure S8. Auditory brainstem response (ABR) recording of No. 19 rat before experiment in salicylate group in Experiment One. Figure S9. Auditory brainstem response (ABR) recording of No. 24 rat before experiment in salicylate group in Experiment One. Figure S10. Auditory brainstem response (ABR) recording of No. 26 rat before experiment in salicylate group in Experiment One. Figure S11. Auditory brainstem response (ABR) recording of No. 11 rat in saline group in Experiment One. Figure S12. Auditory brainstem response (ABR) recording of No. 13 rat in saline group in Experiment One. Figure S13. Auditory brainstem response (ABR) recording of No. 22 rat in saline group in Experiment One. Figure S14. Auditory brainstem response (ABR) recording of No. 28 rat in saline group in Experiment One. Figure S15. Auditory [file pone.0108659.s001.zip › File S1/Figure S6.docx]

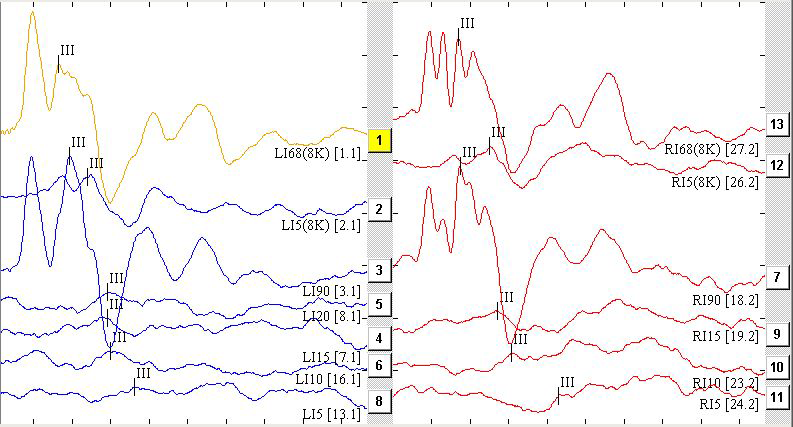


Figure F. Auditory brainstem response (ABR) recording of No. 16 rat before experiment in salicylate group in Experiment One. “L” stands for left ear; “R” stands for right ear; “I” stands for intensity of click or tone; “8K” stands for the frequency of tone is 8000 Hz; “[a. b]”: “a” represents the chronological number of the test and “b” refers to the channel number collected.
