# Supplementary material for: Both Central and Peripheral Auditory Systems Are Involved in Salicylate-Induced Tinnitus in Rats: A Behavioral Study
Source: PLoS One. 2014 Sep 30;9(9):e108659. doi: 10.1371/journal.pone.0108659 (PMC4182535; doi:10.1371/journal.pone.0108659)
Supplement: File S2 — Figure S1. Auditory brainstem response (ABR) recording of No. 55 rat after surgery in Experiment Two. Figure S2. Auditory brainstem response (ABR) recording of No. 56 rat after surgery in Experiment Two. Figure S3. Auditory brainstem response (ABR) recording of No. 57 rat after surgery in Experiment Two. Figure S4. Auditory brainstem response (ABR) recording of No. 55 rat before experiment in Experiment Two. Figure S5. Auditory brainstem response (ABR) recording of No. 56 rat before experiment in Experiment Two. Figure S6. Auditory brainstem response (ABR) recording of No. 57 rat before experiment in Experiment Two. (ZIP) [file pone.0108659.s002.zip › File S2/Figure S4.docx]

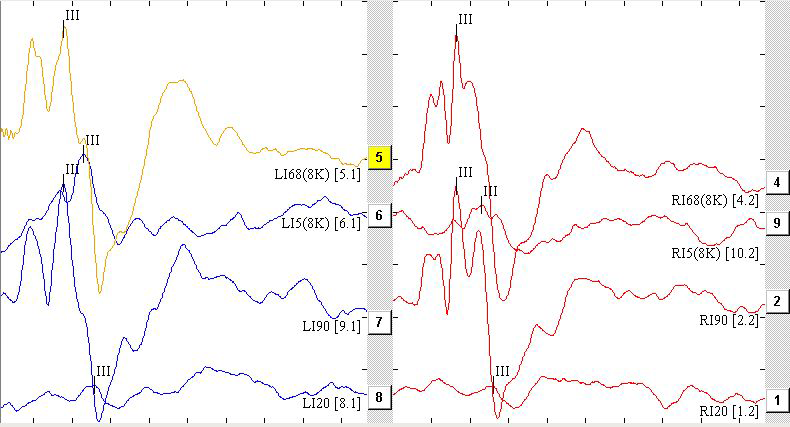


Figure D. Auditory brainstem response (ABR) recording of No. 55 rat before experiment in Experiment Two. “L” stands for left ear; “R” stands for right ear; “I” stands for intensity of click or tone; “8K” stands for the frequency of tone is 8000 Hz; “[a. b]”: “a” represents the chronological number of the test and “b” refers to the channel number collected.
