# Supplementary material for: Both Central and Peripheral Auditory Systems Are Involved in Salicylate-Induced Tinnitus in Rats: A Behavioral Study
Source: PLoS One. 2014 Sep 30;9(9):e108659. doi: 10.1371/journal.pone.0108659 (PMC4182535; doi:10.1371/journal.pone.0108659)
Supplement: File S3 — Figure S1. Auditory brainstem response (ABR) recording of No. 10 rat after surgery in salicylate group in Experiment Three. Figure S2. Auditory brainstem response (ABR) recording of No. 20 rat after surgery in salicylate group in Experiment Three. Figure S3. Auditory brainstem response (ABR) recording of No. 24 rat after surgery in salicylate group in Experiment Three. Figure S4. Auditory brainstem response (ABR) recording of No. 33 rat after surgery in salicylate group in Experiment Three. Figure S5. Auditory brainstem response (ABR) recording of No. 51 rat after surgery in salicylate group in Experiment Three. Figure S6. Auditory brainstem response (ABR) recording of No. 10 rat before experiment in salicylate group in Experiment Three. Figure S7. Auditory brainstem response (ABR) recording of No. 20 rat before experiment in salicylate group in Experiment Three. Figure S8. Auditory brainstem response (ABR) recording of No. 24 rat before experiment in salicylate group in Experiment Three. Figure S9. Auditory brainstem response (ABR) recording of No. 33 rat before experiment in salicylate group in Experiment Three. Figure S10. Auditory brainstem response (ABR) recording of No. 51 rat before experiment in salicylate group in Experiment Three. Figure S11. Auditory brainstem response (ABR) recording of No. 12 rat after surgery in saline group in Experiment Three. Figure S12. Auditory brainstem response (ABR) recording of No. 15 rat after surgery in saline group in Experiment Three. Figure S13. Auditory brainstem response (ABR) recording of No. 21 rat after surgery in saline group in Experiment Three. Figure S14. Auditory brainstem response (ABR) recording of No. 22 rat after surgery in saline group in Experiment Three. Figure S15. Auditory brainstem response (ABR) recording of No. 25 rat after surgery in saline group in Experiment Three. Figure S16. Auditory brainstem response (ABR) recording of No. 12 rat before experiment in saline group in Experiment Three. Figure S1 [file pone.0108659.s003.zip › File S3/Figure S9.docx]

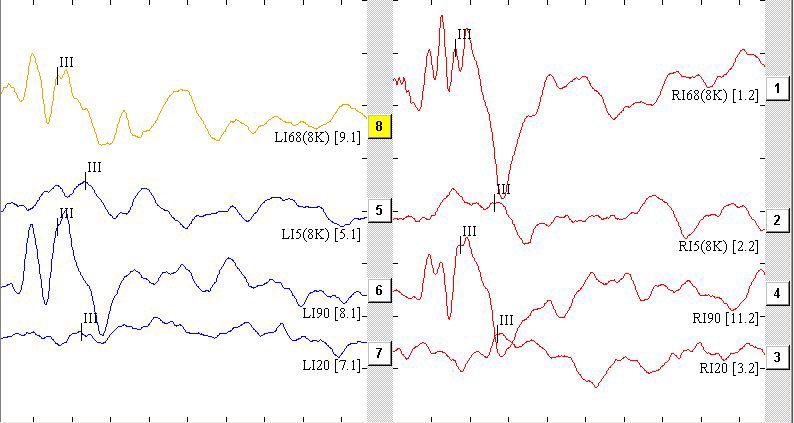


Figure I. Auditory brainstem response (ABR) recording of No. 33 rat before experiment in salicylate group in Experiment Three. “L” stands for left ear; “R” stands for right ear; “I” stands for intensity of click or tone; “8K” stands for the frequency of tone is 8000 Hz; “[a. b]”: “a” represents the chronological number of the test and “b” refers to the channel number collected.
